# Supplementary material for: Larval dispersal of Brachyura in one of the largest estuarine/marine systems in the world
Source: PLoS One. 2022 Aug 25;17(8):e0252695. doi: 10.1371/journal.pone.0252695 (PMC9410557; doi:10.1371/journal.pone.0252695)
Supplement: S1 Table — (DOCX) [file pone.0252695.s009.docx]

**S9. Descriptive statistical (minimum, maximum, mean and standard deviation) of environmental parameters (temperature, salinity and chlorophyll-a) in relation to months and distance from the coast on the Amazon Continental Shelf.**

|  |  | **Jul/13** | | | **Oct/13** | **Jan/14** | | | **May/14** | **Jul/14** | | | **Oct/14** | **Jan/15** |
| --- | --- | --- | --- | --- | --- | --- | --- | --- | --- | --- | --- | --- | --- | --- |
| Temp (°C) | Minimum | | 25.67 | 26.16 | | | 22.96 | 23.12 | | | 27.20 | 23.39 | | 27.24 |
|  | Maximum | | 29.85 | 29.17 | | | 28.77 | 29.01 | | | 29.12 | 29.23 | | 28.98 |
|  | Mean ± sd | | 28.34 ± 0.63 | 27.87 ± 0.61 | | | 27.44 ± 0.93 | 27.73 ± 1.18 | | | 28.32 ± 0.62 | 27.35 ± 1.06 | | 27.94 ± 0.52 |
| Salinity | Minimum | | 3.57 | 19.97 | | | 16.23 | 2.54 | | | 4.64 | 17.80 | | 10.37 |
|  | Maximum | | 37.01 | 37.60 | | | 37.25 | 37.16 | | | 37.52 | 37.71 | | 37.73 |
|  | Mean ± sd | | 35.11 ± 4.81 | 36.28 ± 2.95 | | | 35.06 ± 5.36 | 30.93 ± 8.88 | | | 35.53 ± 4.94 | 37.02 ± 2.79 | | 36.04 ± 4.61 |
| Chlorophyll-*a* | Minimum | | 2.14 | 1.60 | | | 0.30 | 3.61 | | | 1.67 | 1.83 | | 0.56 |
|  | Maximum | | 52.76 | 43.72 | | | 85.10 | 31.45 | | | 37.64 | 16.56 | | 29.34 |
|  | Mean ± sd | | 15.24 ± 11.52 | 7.78 ± 8.64 | | | 10.5 ± 17.10 | 6.48 ± 3.89 | | | 7.40 ± 7.41 | 4.67 ± 2.46 | | 3.93 ± 4.59 |

|  |  | | **23 Km** | **53 Km** | | | **83 Km** | **158 Km** | | | **198 Km** | **233 Km** | |
| --- | --- | --- | --- | --- | --- | --- | --- | --- | --- | --- | --- | --- | --- |
| Temp (°C) | Minimum | 28.35 | | | 28.08 | 27.99 | | | 27.08 | 27.06 | | | 22.96 |
|  | Maximum | 29.12 | | | 29.85 | 29.34 | | | 28.76 | 28.89 | | | 28.59 |
|  | Mean ± sd | 28.65 ± 0.26 | | | 28.72 ± 0.37 | 28.62 ± 0.36 | | | 28.0 ± 0.54 | 27.66 ± 0.45 | | | 27.22 ± 1.10 |
| Salinity | Minimum | 3.57 | | | 2.54 | 6.13 | | | 16.05 | 25.71 | | | 33.26 |
|  | Maximum | 24.93 | | | 37.34 | 37.73 | | | 37.71 | 37.69 | | | 37.70 |
|  | Mean ± sd | 16.73 ± 5.97 | | | 29.09 ± 8.89 | 34.49 ± 5.50 | | | 36.23 ± 3.21 | 36.77 ± 1.45 | | | 37.03 ± 0.57 |
| Chlorophyll-*a* | Minimum | 4.88 | | | 7.08 | 3.06 | | | 1.62 | 1.72 | | | 0.30 |
|  | Maximum | 45.69 | | | 85.11 | 39.35 | | | 52.76 | 43.74 | | | 36.32 |
|  | Mean ± sd | 25.92 ± 11.78 | | | 19.95 ± 17.95 | 10.3 ± 17.35 | | | 6.44 ± 8.17 | 6.20 ± 5.88 | | | 4.29 ± 3.56 |
