# Supplementary material for: Larval dispersal of Brachyura in one of the largest estuarine/marine systems in the world
Source: PLoS One. 2022 Aug 25;17(8):e0252695. doi: 10.1371/journal.pone.0252695 (PMC9410557; doi:10.1371/journal.pone.0252695)
Supplement: S2 Table — “SS” is used as an abbreviation for sub-superficial sample and "O" to oblique hauls. (DOCX) [file pone.0252695.s010.docx]

**S10. Larval composition, N total and sum of density (larvae m^-3^, in parenthesis) of Brachyura in each sampling sites on the Amazon Continental Shelf. “SS” is used as an abbreviation for sub-superficial sample and "O" to oblique hauls.**

| **Taxon** | **Stages/phase** | **Hauls** | **23km** | **53km** | **83km** | **158km** | **198km** | **233km** |
| --- | --- | --- | --- | --- | --- | --- | --- | --- |
| **Calappidae** |  |  |  |  |  |  |  |  |
| *Calappa* sp. | ZI–ZIV | SS | - | - | 4 (0.043) | 2 (0.005) | 2 (0.006) | - |
|  | ZI–ZIV | O | - | - | 8 (0.022) | 188 (0.50) | 6 (0.024) | 72 (0.14) |
| **Grapsidae** |  |  |  |  |  |  |  |  |
| *Goniopsis cruentata* | ZI | SS | 2 (0.009) | - | - | - | - | - |
|  | ZI | O | 12 (0.043) | - | - | - | - | - |
| Grapsidae n. id. | ZII–ZIV | SS | - | - | - | - | - | - |
|  | ZII–ZIV | O | - | - | 2 (0.005) | 4 (0.006) | 4 (0.018) | 12 (0.023) |
| *Pachygrapsus gracilis* | ZI–ZIII | SS | 12 (0.053) | 2 (0.007) | 20 (0.062) | 16 (0.07) | 10 (0.036) | 14 (0.08) |
|  | ZI–ZIII | O | 66 (0.272) | 8 (0.032) | 56 (0.167) | 10 (0.017) | 16 (0.066) | 8 (0.015) |
| **Leucosiidae** |  |  |  |  |  |  |  |  |
| Leucosiidae n. id. | ZII–ZIV | SS | - | - | - | - | 10 (0.032) | - |
|  | ZII–ZIV | O | - | - | - | 4 (0.006) | 2 (0.009) | 4 (0.001) |
| *Persephona* spp. | ZI–ZIV | SS | - | 2 (0.003) | 4 (0.003) | - | - | 2 (0.003) |
|  | ZI–ZIV | O | - | 2 (0.006) | 18 (0.039) | 4 (0.006) | 2 (0.009) | 6 (0.008) |
| **Ocypodidae** |  |  |  |  |  |  |  |  |
| Gelasiminae 1 | ZI | SS | 122 (1.04) | 4 (0.011) | 2 (0.021) | - | - | - |
|  | ZI | O | - | - | - | 2 (0.006) | - | - |
| Gelasiminae 2 | ZI | SS | 262 (2.01) | 18 (0.101) | 8 (0.087) | 2 (0.005) | 2 (0.007) | 2 (0.004) |
|  | ZI | O | 398 (2.28) | 24 (0.09) | 4 (0.019) | 8 (0.024) | 2 (0.025) | - |
| Gelasiminae 3 | ZI | SS | 88 (0.686) | 4 (0.022) | 2 (0,021) | - | - | - |
|  | ZI | O | 38 (0.278) | - | - | - | - | - |
| Gelasiminae n. id. | ZII–ZVI | SS | 10 (0.111) | 134 (3.57) | 550 (4.34) | 20 (0.094) | - | 2 (0.004) |
|  | ZII–ZVI | O | 10 (0.121) | 28 (0.36) | 170 (0.897) | 6 (0.018) | 2 (0.009) |  |
| *Leptuca cumulanta* | ZI. ZII | SS | 2 (0.009) | - | - | - | - | - |
|  | ZI. ZII | O | 4 (0.014) | - | - | - | - | - |
| Megalopa 1 |  | SS | 8 (0.041) | 10 (0.27) | 28 (0.22) | - | - | - |
|  |  | O | - | - | 56 (0.30) | - | - | - |
| Megalopa 2 |  | SS | 2 (0.007) | - | - | - | - | - |
|  |  | O | - | - | - | - | - | - |
| *Minuca rapax* | ZI, ZIII, ZIV | SS | 64 (0.547) | 12 (0.218) | 6 (0.059) | - | - | - |
|  | ZI, ZIII, ZIV | O | 20 (0.349) | 10 (0.062) | - | - | - | - |
| *Uca maracoani* | ZI, ZIII, ZIV | SS | - | 4 (0.011) | 2 (0.006) | - | - | - |
|  | ZI, ZIII, ZIV | O | - | 2 (0.034) | 4 (0.012) | - | - | - |
| *Ucides cordatus* | ZI | SS | 6 (0.031) | - | - | - | - | - |
|  | ZI | O | - | - | - | - | - | - |
| **Panopeidae** |  |  |  |  |  |  |  |  |
| *Hexapanopeus* spp. | ZI–ZIV | SS | 10 (0.064) | 12 (0.034) | 54 (0.117) | - | - | - |
|  | ZI–ZIV | O | - | 10 (0.036) | 50 (0.107) | 2 (0.004) | - | - |
| Megalopa |  | SS | - | - | 8 (0.006) | - | - | 2 (0.0007) |
|  |  | O | - | - | - | - | - |  |
| *Panopeus lacustris* | ZI–ZIV, M | SS | 40 (0.258) | 1590 (10.41) | 1136 (8.84) | 48 (0.356) | - | 17152 (46.88) |
|  | ZI–ZIV, M | O | 4 (0.094) | 1246 (6.66) | 946 (4.95) | 124 (0.30) | 118 (0.467) | 2706 (6.52) |
| *Panopeus* sp. | ZI–ZIV | SS | 4 (0.019) | 28 (0.08) | 2 (0.006) | 2 (0.040) | - | 12 (0.034) |
|  | ZI–ZIV | O | - | 4 (0.015) | - | - | - | 12 (0.023) |
| **Pinnotheridae** |  |  |  |  |  |  |  |  |
| *Austinixa* sp. | ZI–ZV | SS | 2 (0.012) | 22 (0.064) | 78 (0.16) | 2 (0.015) | - | - |
|  | ZI–ZV | O | - | 54 (0.207) | 66 (0.14) | 4 (0.009) | - | - |
| *Dissodactylus crinitichelis* | ZI–ZIV | SS | - | - | 244 (0.20) | 10 (0.031) | - | - |
|  | ZI–ZIV | O | - | - | 62 (0.067) | 14 (0.011) | - | - |
| *Pinnixa* sp. | ZI–ZV | SS | 34 (0.251) | 64 (0.59) | 44 (0.414) | - | 2 (0.006) | 2 (0.005) |
|  | ZI–ZV | O | 88 (0.553) | 108 (0.71) | 54 (0.252) | 4 (0.012) | - | - |
| Megalopa |  | SS | - | - | - | - | - | - |
|  |  | O | - | - | 2 (0.001) | - | - | - |
| **Portunidae** |  |  |  |  |  |  |  |  |
| *Achelous* spp. | ZI–ZVII | SS | 6 (0.031) | 32 (0.073) | 672 (5.09) | 278 (1.90) | 252 (0.88) | 796 (1.72) |
|  | ZI–ZVII | O | 2 (0.007) | 36 (0.116) | 686 (1.55) | 1158 (2.24) | 210 (1.04) | 516 (1.03) |
| *Callinectes* spp. | ZI–ZVIII | SS | - | - | 20 (0.016) | 118 (1.16) | 6 (0.018) | 160 (0.33) |
|  | ZI–ZVIII | O | - | 6 (0.018) | 72 (0.261) | 302 (0.56) | 10 (0.027) | 40 (0.079) |
| Megalopa 1 |  | SS | 2 (0.010) | - | 10 (0.033) | 2 (0.015) | - | - |
|  |  | O | - | - | - | 4 (0.004) | - | 6 (0.012) |
| Megalopa 2 |  | SS | - | - | - | 2 (0.002) | - | - |
|  |  | O | - | - | - |  | - | 2 (0.01) |
| Portunidae n. id. | ZI–ZIII | SS | - | - | 4 (0.015) | 2 (0.005) | 4 (0.012) | 24 (0.049) |
|  | ZI–ZIII | O | - | - | - | 14 ( 0.021) | - | - |
| **Sesarmidae** |  |  |  |  |  |  |  |  |
| *Armases rubripes* | ZI–ZIV, M | SS | 486 (3.74) | 1744 (40.34) | 72 (0.878) | 6 (0.028) | - | - |
|  | ZI–ZIV, M | O | 284 (3.79) | 482 (5.77) | 208 (1.19) | - | - | 4 (0.009) |

ZI = zoea I; ZII = zoea II; ZIII = zoea III; ZIV = zoea IV; ZV = zoea V; ZVI = zoea VI; ZVII = zoea VII; ZVIII = zoea VIII; M = megalopa; n. id. = not identified.
