# Supplementary material for: Larval dispersal of Brachyura in one of the largest estuarine/marine systems in the world
Source: PLoS One. 2022 Aug 25;17(8):e0252695. doi: 10.1371/journal.pone.0252695 (PMC9410557; doi:10.1371/journal.pone.0252695)
Supplement: S3 Table — “SS” is used as an abbreviation for sub-superficial sample and "O" to oblique hauls. (DOCX) [file pone.0252695.s011.docx]

**S11. Larval composition, N total and sum of density (larvae m^-3^ , in parenthesis) of Brachyura in each expedition on the Amazon Continental Shelf. “SS” is used as an abbreviation for sub-superficial sample and "O" to oblique hauls**.

| **Taxon** | **Stages/phase** | **Hauls** | **Jul 13** | **Oct 13** | **Jan 14** | **May 14** | **Jul 14** | **Oct 14** | **Jan 15** |
| --- | --- | --- | --- | --- | --- | --- | --- | --- | --- |
| **Calappidae** |  |  |  |  |  |  |  |  |  |
| *Calappa* sp. | ZI–ZIV | SS | - | - | - | 6 (0.049) | - | 2 (0.005) | - |
|  | ZI–ZIV | O | 12 (0.02) | 2 (0.004) | 34 (0.10) | 104 (0.25) | 2 (0.004) | 114 (0.29) | 6 (0.0014) |
| **Grapsidae** |  |  |  |  |  |  |  |  |  |
| *Goniopsis cruentata* | ZI | SS | - | 2 (0.009) | - | - | - | - | - |
|  | ZI | O | - | 12 (0.04) | - | - | - | - | - |
| Grapsidae n. id. | ZII–ZIV | SS | - | - | - | - | - | - | - |
|  | ZII–ZIV | O | - | - | 2 (0.005) | 12 (0.023) | 4 (0.018) | 2 (0.005) | 2 (0.0001) |
| *Pachygrapsus gracilis* | ZI–ZIII | SS | 2 (0.054) | 18 (0.081) | - | 16 (0.037) | 4 (0.012) | 26 (0.086) | 8 (0.042) |
|  | ZI–ZIII | O | 4 (0.017) | 66 (0.223) | 6 (0.027) | 8 (0.015) | 18 (0.079) | 60 (0.206) | 2 (0.0001) |
| **Leucosiidae** |  |  |  |  |  |  |  |  |  |
| Leucosiidae n. id. | ZII–ZIV | SS | - | - | 4 (0.011) | - | 4 (0.014) | 2 (0.0071) | - |
|  | ZII–ZIV | O | - | 4 (0.0063) | - | - | 2 (0.0091) | - | 4 (0.0014) |
| *Persephona* spp. | ZI–ZIV | SS | - | - | - | - | 2 (0.0037) | - | 6 (0.0064) |
|  | ZI–ZIV | O | - | 16 (0.029) | - | 4 (0.007) | - | 8 (0.022) | 4 (0.0098) |
| **Ocypodidae** |  |  |  |  |  |  |  |  |  |
| Gelasiminae 1 | ZI | SS | - | 6 (0.021) | - | 120 (1.46) | - | 2 (0.012) | - |
|  | ZI | O | - | - | - | 2 (0.006) | - | - | - |
| Gelasiminae 2 | ZI | SS | 24 (0.166) | 48 (0.229) | - | 206 (1.738) | - | 16 (0.089) | - |
|  | ZI | O | - | 66 (0.241) | 2 (0.047) | 48 (0.698) | 2 (0.007) | - | 318 (1.452) |
| Gelasiminae 3 | ZI | SS | - | 20 (0.095) | - | 74 (0.634) | - | - | - |
|  | ZI | O | - | 28 (0.10) | - | 10 (0.176) | - | - | - |
| Gelasiminae n. id. | ZII–ZVI | SS | 4 (0.049) | 6 (0.021) | 672 (7.93) | 24 (0.10) | 10 (0.001) | - | - |
|  | ZII–ZVI | O | 10 (0.059) | 14 (0.048) | 178 (1.24) | 6 (0.018) | 2 (0.008) | 2 (0.012) | 4 (0.013) |
| *Leptuca cumulanta* | ZI, ZII | SS | - | 2 (0.009) | - | - | - | - | - |
|  | ZI, ZII | O | - | 4 (0.014) | - | - | - | - | - |
| Megalopa 1 |  | SS | 2 (0.024) | 6 (0.028) | 36 (0.46) | - | - | 2 (0.012) | - |
|  |  | O | - | - | 56 (0.30) | - | - | - | - |
| Megalopa 2 |  | SS | - | - | - | - | 2 (0.007) | - | - |
|  |  | O | - | - | - | - | - | - | - |
| *Minuca rapax* | ZI, ZIII, ZIV | SS | 0 | 2 (0.009) | 8 (0.19) | 72 (0.61) | - | - | - |
|  | ZI, ZIII, ZIV | O | 0 | 2 (0.007) | 4 (0.094) | 24 (0.31) | - | - | - |
| *Uca maracoani* | ZI, ZIII, ZIV | SS | 0 | 4 (0.011) | - | - | - | 2 (0,006) | - |
|  | ZI, ZIII, ZIV | O | 6 (0.046) | - | - | - | - | - | - |
| *Ucides cordatus* | ZI | SS | - | - | - | - | - | - | 6 (0.031) |
|  | ZI | O | - | - | - | - | - | - | - |
| **Panopeidae** |  |  |  |  |  |  |  |  |  |
| *Hexapanopeus* spp. | ZI–ZIV | SS | 8 (0.055) | 14 (0.043) | 8 (0.063) | - | 4 (0.020) | - | 42 (0.033) |
|  | ZI–ZIV | O | - | 32 (0.076) | 8 (0.043) | - | - | 4 (0.011) | 18 (0.017) |
| Megalopa |  | SS | - | - | - | - | - | - | 10 (0.006) |
|  |  | O | - | - | - | - | - | - | 2 (0.0007) |
| *Panopeus lacustris* | ZI–ZIV, M | SS | 52 (1.096) | 17762 (48.28) | 1444 (15.8) | 10 (0.084) | 560 (0.082) | 78 (0.29) | 60 (0.36) |
|  | ZI–ZIV, M | O | 118 (0.41) | 2902 (7.54) | 1132 (7.90) | 50 (0.14) | 342 (1.24) | 520 (1.65) | 80 (0.12) |
| *Panopeus* sp. | ZI–ZIV | SS | 2 (0.04) | 34 (0.10) | 8 (0.023) | - | - | 4 (0.011) | - |
|  | ZI–ZIV | O | - | 4 (0.015) | - | 12 (0.023) | - | - | - |
| **Pinnotheridae** |  |  |  |  |  |  |  |  |  |
| *Austinixa* sp. | ZI–ZV | SS | - | 20 (0.057) | 14 (0.11) | - | - | 4 (0.058) | 66 (0.066) |
|  | ZI–ZV | O | 6 (0.018) | 40 (0.12) | 10 (0.083) | 4 (0.018) | - | 10 (0.027) | 54 (0.091) |
| *Dissodactylus crinitichelis* | ZI–ZIV | SS | - | - | - | - | 12 (0.041) | - | 242 (0.19) |
|  | ZI–ZIV | O | - | 4 (0.006) | - | - | 4 (0.004) | 4 (0.011) | 64 (0.057) |
| *Pinnixa* sp. | ZI–ZV | SS | 10 (0.19) | 48 (0.15) | 58 (0.81) | 2 (0.006) | 10 (0.011) | 12 (0.071) | 6 (0.013) |
|  | ZI–ZV | O | 6 (0.018) | 92 (0.34) | 50 (0.67) | 18 (0.093) | 10 (0.041) | 54 (0.03) | 24 (0.053) |
| Megalopa |  | SS | - | - | - | - | - | - | - |
|  |  | O | - | - | - | - | - | - | 2 (0.019) |
| **Portunidae** |  |  |  |  |  |  |  |  |  |
| *Achelous* spp. | ZI–ZVII | SS | 114 (4.67) | 188 (0.71) | 66 (0.31) | 980 (2.31) | 228 (0.89) | 48 (0.16) | 412 (0.63) |
|  | ZI–ZVII | O | 698 (1.74) | 466 (0.86) | 110 (0.36) | 582 (1.39) | 332 (0.98) | 178 (0.51) | 242 (0.14) |
| *Callinectes* spp. | ZI–ZVIII | SS | 46 (0.93) | - | - | 162 (0.34) | 26 (0.077) | 48 (0.143) | 22 (0.031) |
|  | ZI–ZVIII | O | 40 (0.097) | 66 (0.11) | 94 (0.278) | 40 (0.082) | 164 (0.35) | 6 (0.018) | 20 (0.003) |
| Megalopa 1 |  | SS | - | - | - | - | 6 (0.03) | - | 8 (0.029) |
|  |  | O | - | - | - | 4 (0.007) | 6 (0.009) | - | - |
| Megalopa 2 |  | SS | - | - | - | - | - | - | - |
|  |  | O | - | - | 2 (0.010) | - | 2 (0.0023) | - | - |
| Portunidae n. id. | ZI–ZIII | SS | - | - | - | 24 (0.054) | 4 (0.007) | 2 (0.005) | - |
|  | ZI–ZIII | O | 4 (0.009) | - | 4 (0.011) | - | 4 (0.015) | 0 | 6 (0.0003) |
| **Sesarmidae** |  |  |  |  |  |  |  |  |  |
| *Armases rubripes* | ZI–ZIV, M | SS | 244 (1.95) | 74 (0.35) | 1348 (39.7) | 302 (2.26) | 20 (0.057) | 6 (0.038) | 314 (0.588) |
|  | ZI–ZIV, M | O | 58 (0.90) | 70 (0.25) | 278 (5.68) | 372 (3.20) | 44 (0.28) | 16 (0.085) | 140 (0.34) |

ZI = zoea I; ZII = zoea II; ZIII = zoea III; ZIV = zoea IV; ZV = zoea V; ZVI = zoea VI; ZVII = zoea VII; ZVIII = zoea VIII; M = megalopa; n. id. = not identified.
