# Supplementary material for: Larval dispersal of Brachyura in one of the largest estuarine/marine systems in the world
Source: PLoS One. 2022 Aug 25;17(8):e0252695. doi: 10.1371/journal.pone.0252695 (PMC9410557; doi:10.1371/journal.pone.0252695)
Supplement: S4 Table — The values vary from 0 (never important) to 1 (always important). PO: Probability of Occurrence; A: Expected Abundance. FND: Fishing Net Depth; S: Salinity; D: Distance from the coast; C: Chlorophyll-a; T: Temperature. The number “two” in their corresponding superscript stands for the variable squared value. (DOCX) [file pone.0252695.s012.docx]

**S12. Variable influence on the predicted abundances (sum of the corresponding Akaike weights) across plausible models (AIC difference threshold less or equal to 2). The values vary from 0 (never important) to 1 (always important). PO: Probability of Occurrence; A: Expected Abundance. FND: Fishing Net Depth; S: Salinity; D: Distance from the coast; C: Chlorophyll-a; T: Temperature. The number “two” in their corresponding superscript stands for the variable squared value.**

|  |  | **FND** | **S** | **D** | **C** | **T** | **S^2^** | **D^2^** | **C^2^** | **T^2^** |
| --- | --- | --- | --- | --- | --- | --- | --- | --- | --- | --- |
| Sesarmidae | PO | 0.42 | 0.42 | 0.42 | 0.167 | 0.034 | 0.028 | 0.385 | 0.111 | 0.115 |
|  | A | 0.94 | 0.94 | 0.94 | 0.94 | 0.94 | 0.94 | 0.94 | 0.94 | 0.94 |
| Pinnotheridae | PO | 0.043 | 0.081 | 0.395 | 0.031 | 0.283 | 0.395 | 0.107 | 0.058 | 0.161 |
|  | A | 1 | 0.303 | 1 | 1 | 1 | 1 | 1 | 1 | 1 |
| Calappidae | PO | 0.315 | 0.156 | 0.315 | 0.045 | 0.084 | 0.182 | 0.315 | 0.021 | 0.087 |
|  | A | 0.712 | 0.712 | 0.252 | 0.712 | 0.712 | 0.712 | 0.712 | 0.136 | 0.712 |
| Grapsidae | PO | 0.007 | 0.147 | 0.123 | 0.032 | 0.033 | 0.038 | 0.056 | 0.102 | 0.016 |
|  | A | 0.282 | 0.882 | 0.882 | 0.882 | 0.882 | 0.445 | 0.882 | 0.882 | 0.882 |
| Leucosiidae | PO | 0.02 | 0.192 | 0.019 | 0.021 | 0.02 | 0.192 | 0.02 | 0.02 | 0.021 |
|  | A | 0.095 | 0.454 | 0.083 | 0.454 | 0.1 | 0.454 | 0.117 | 0.454 | 0.043 |
| Ocypodidae | PO | 0.026 | 0.182 | 0.287 | 0.04 | 0.112 | 0.153 | 0.027 | 0.025 | 0.238 |
|  | A | 1 | 1 | 1 | 1 | 1 | 1 | 1 | 1 | 1 |
| Panopeidae | PO | 0.02 | 0.351 | 0.064 | 0.351 | 0.047 | 0.064 | 0.089 | 0.141 | 0.185 |
|  | A | 1 | 1 | 1 | 1 | 1 | 1 | 1 | 1 | 1 |
| Portunidae | PO | 0.23 | 0.23 | 0.026 | 0.047 | 0.03 | 0.013 | 0.23 | 0.1 | 0.061 |
|  | A | 0.881 | 0.881 | 0.881 | 0.881 | 0 | 0.881 | 0.881 | 0.881 | 0.881 |
