# Supplementary material for: Larval dispersal of Brachyura in one of the largest estuarine/marine systems in the world
Source: PLoS One. 2022 Aug 25;17(8):e0252695. doi: 10.1371/journal.pone.0252695 (PMC9410557; doi:10.1371/journal.pone.0252695)
Supplement: Attachment — Submitted filename: PONE-D-21-16420_review.pdf [file pone.0252695.s013.pdf]

PONE-D-21-16420
Reviewer Comments

This study summarizes field distributions of Brachyuran crab larvae along the Amazon Continental Shelf. The authors describe larval dispersal of diverse taxa across both spatial (depth/distance offshore) and seasonal scales. To date, relatively few studies have reported extensive field distributions of Brachyura during larval development. Hence, this study provides a valuable contribution to better understanding their dispersal patterns.

Please find my comments and suggestions below. Many of my comments are minor suggestions, although there are four major concerns that I hope the authors will address in revision. First, the phrase “larval dispersion” is used incorrectly and should most likely be replaced with “larval dispersal” throughout the manuscript. In ecology, dispersion indicates investigation of specific distribution patterns, e.g. random, clumped, or uniform, whereas “dispersal” indicates the movement of individuals, e.g. export vs. retention. Second, the figures that show model results (Figs. 3 – 5) are missing key information, which make it difficult to confirm the authors’ interpretation of the data. I provided specific comments to this regard below. Third, the paper should include a summary of model outcomes in the results section to support statements made later in the discussion. For example, the authors state that salinity was a reliable predictor of larval dispersal, while temperature and chlorophyll-a were not; however, statistical support for this claim is lacking. Overall, I find it concerning that an extensive multi-model approach was described in the methods section, but there is little description of the outcomes in the results section (aside from Figs. 3 – 5, which require more detail in their respective captions). Fourth, the impact of the seasonal plume is a central point in the paper. However, statistical/graphical support is lacking (see my comment regarding lines 285 – 297 below). The authors reference Figs. 6 and 7 to support this analysis. However, these figures were not included in this submission.

In summary, this study represents a valuable contribution to better understanding the dispersal of larval Brachyurans in coastal systems. However, I recommend that the concerns presented in this review be addressed prior to publication.

Abstract

The importance of the Amazon River seasonal flow and plume is not mentioned until the end of the abstract. Opening the abstract with a sentence or two describing the system would help guide the reader through the results that are summarized just below.

Lines 27 – 31: This sentence runs on a bit. It would be useful to more clearly highlight the two objectives described: (1) to analyze the composition of larval Brachyuran crabs and (2) to predict the importance of environmental parameters in structuring their occurrence/abundance.

Line 31: “A total of 17,759 identified larvae are…” should be “A total of 17,759 identified larvae were…”

Line 39: “(> = 33.5)” should be “(≥ 33.5)”

Line 45: Remove “plankton”

Introduction

Line 73: Do you mean “harbors”, rather than “habirs” here?

Line 81: Why include “among others”?

Lines 99 – 103: I suggest restructuring of this sentence. It might be useful to break it up into (1) parameters of larval composition/distribution and (2) how these relate to environmental profiles. The current organization is a bit convoluted.

Line 104: The “aquatic food chain” is an oversimplification. Consider using “food web” or “trophic interactions” instead.

Line 108: I’m not sure what is meant by “…should be distributed on their parental populations…” Are you expecting close proximity to parental populations? Given the common export strategy of estuarine crabs, would this be likely for all Brachyuran crabs in the region?

Methods

Lines 115 – 116: “July/2013 to January/2015” should be “July 2013 to January 2015”

Line 131: “haulsin” should be “hauls in”

Lines 154 – 155: The density unit should have not a period after larvae, and the sentence could be restructured for clarity, e.g. “Density (larvae m^{-3}) was estimated by dividing Brachyuran larval abundance by the volume filtered through the plankton net.”

Line 161: “…of each species larvae…” should be “…of each larval species…” or “…of each species of larvae”

Line 200: Citation(s) to support that this threshold is “widely used”?

Results

Line 208 and 212: Replace “amplitude” with “range”

Line 209: The parenthetical “begin” should be “beginning” in both occurrences here.

Line 217: Remove the “/” between month and year

Line 248: Add “and” before *Pinnixa*

Line 285 – 297: I think that the seasonal plume analysis discussed here is important. However, this section requires visual, model, and/or statistical support. Figure 6 and 7 are referenced in this paragraph but these were not included in the submission. In addition, several of the statements made cannot be supported by Table 1 or Figs. 3 – 5, which do not include seasonal information.

Discussion

Lines 301 – 303: To make this statement, more support is needed in the results section (see previous comment regarding lines 285 – 297).

Line 311: Should be “Brachyuran larvae”

Lines 320 – 323: Where are the model results that support this statement?

Line 470: Remove “Anyway”

Line 515: Is “particular” the best word here?

Line 531: Why change the subtitle structure at this point? All others list the family only.

Line 561: Same as above – why alter the subtitle structure?

Line 563: “larval” before zoea is a bit redundant

Line 585: Either add a semicolon after “…(Herbst, 1803)” or start a new sentence, i.e. “The latter is…” The structure of species name reporting also switches to parentheticals here: “*Calappa gallus* (Herbst, 1803)” rather than the previous “*Calappa sulcata* Rathbun, 1898”. For consistency, use the same format throughout.

Line 661: “And” should not be capitalized.

Table and Figures

Table 1 only shows the frequency of occurrence for one month/year for each group. Based on the supplemental figures, I assume this is the timepoint with the highest density for each individual taxon. If so, indicate this in the table caption. I also suggest characterizing the colors of the heat map described, i.e. frequency of occurrence increases in order of white, light gray,

dark gray, and black. Also, “S” is used as an abbreviation for salinity and for sub-superficial sample. Perhaps, it would be clearer to change the latter to “SS”.

Fig. 1 caption: It would be useful to identify that the distances listed are kilometers offshore, e.g. “…(23 km, 53 km, 83 km, 158 km, 198 km, and 233 km offshore).”

Figs. 3 – 5: More information is needed in the caption, which could also be accomplished by adding a legend. For example, what do the different colors represent – different models? Do the shaded regions around each trendline indicate a confidence interval of some sort? Are the y-axis values shown expected abundance per some unit of volume? The methods state that the “final prediction for the expected abundance of each group is given by the product between its predicted abundance and the PO in the reference scenario”. However, the notation in the y-axis label indicates that you are showing a ratio of predicted abundance / PO, rather than the product.

Supporting information

S9 and S10 were not referenced in the text.
