# Supplementary material for: Larval dispersal of Brachyura in one of the largest estuarine/marine systems in the world
Source: PLoS One. 2022 Aug 25;17(8):e0252695. doi: 10.1371/journal.pone.0252695 (PMC9410557; doi:10.1371/journal.pone.0252695)
Supplement: Attachment — Submitted filename: Response to Reviewers.docx [file pone.0252695.s015.docx]

We responded to the suggestions of referees, and the answers to each point are highlighted in blue color.

**Editor: General comments**

There is just one (really minor!) point, where I do not agree with a change of phrasing suggested by Rev. #2: "Line 176: “...of each species larvae...” should be “...of each larval species...” or “...of each species of larvae”. - Answer: The sentence “...of each species larvae” was changed for “...of each larval species".

The original phrasing was in fact a little bit awkward in style (actually, only an apostrophe was lacking after species’), but it was semantically unambiguous. In my opinion, a "larval species" (as suggested by the reviewer and adopted by the authors) would be something like an axolotl, i.e. a species that retains a larval form throughout its life. This is not the case in crabs. - Similarly, a "species of larvae" does not exist. There are only larvae that are described and assigned to an already known species (or higher taxon) that had previously been described as an adult form. The description of larvae as supposedly different species occurred typically in the 19th century when complex life histories were largely unknown.

If the Editors and the authors agree with me, I suggest to rephrase p. 8, Line 176 as follows: "The frequency of larval occurrence (FO) was given for each species as a x 100/A, where ..."

Answer: Yes, we agree. We agree with the editor's general comments “The original phrasing was, in fact, a little bit awkward in style (actually, only an apostrophe was lacking after species’), but it was semantically unambiguous. The sentence was restructured: "The frequency of larval occurrence (FO %) was given for each species as FO = a × 100/A, where a = number of samples containing the species, and A = the total number of samples." Thanks for your valuable suggestion.

**Reviewer #2: General comments**

The authors have made a significant effort to revise their original manuscript, including satisfactory responses to my major concerns. The revision also benefits from a clearer description of the sampling protocol and a more concise discussion section. Below, I describe minor suggestions for final revision of this manuscript.

Answer: We are grateful for the reviewer's thorough attention and observations/notes and we hope this paper is ready to be published in Plos One.

Suggestions for Improvements

Line 62: “Dispersion” is used but I think the authors are commenting on “dispersal” strategies, i.e., larval transport rather than distribution patterns.

Answer: The word “dispersion” was substituted for “dispersal”. Only in the last paragraph of the discussion the word “dispersion" was maintained because it deals with distribution patterns. Thanks for the suggestion.

Line 103: “analised” should be “analyzed”

Answer: The word was corrected. Thanks.

Lines 150 – 153: Was the CTD probe attached to the plankton net or were vertical CTD casts done to a particular depth just before or after plankton collection? It is more common to deploy vertical CTD casts just prior to plankton collection, while the ship is stationary. However, the authors state that the CTD “was used to get oblique samples”, implying that the CTD probe was towed.

Answer: Before plankton collection the CTD deploys vertical casts while the ship is stationary. The text was misspelled and we changed it to: Before plankton samples, the environmental variables water temperature (ºC), salinity, and chlorophyll-a (µg / L) contents were obtained using a CTD probe (Hydrolab DS 5) that was used to get samples every 0.5 meters. Oblique was one of the plankton trawlers, not the CTD.

Line 325: *Pinnixa* sp. is cited here but this species’ family (Pinnotheridae) is shown in Figure 6B. Why refer to only one species in this family, when the visual evidence provided summarizes the entire family?

Answer: Only *Pinnixa* sp. was inserted because *Austinixa* occurs only up to 158km and *D. crinitichelis* only close to the GARS areas, and does not vary regarding the plume. We cannot generalize this conclusion to *D. Crinitichelis* because it had a very specific occurrence.

Lines 325 – 330: This statement suggests that larvae in families represented by Fig. 6A, 6B, 6C, 6D, and 7H were concentrated in the mid and outer continental shelf during time of greatest discharge (May) and in coastal areas during time of lowest discharge (October). I’m having a hard time seeing this trend in Fig. 7H (Leucosiidae); the greatest density appears to be in the mid continental shelf (158 km) in October.

Answer: The Reviewer is correct. Leucosiidae density is higher on the mid continental shelf (158 km), while only Grapsidae (6A), Panopeidae (6B), Pinnotheridae (6C), and Portunidae (6D) follow the freshwater plume. We restructure as follows:
“During the highest flow period, Pinnixa sp., Grapsidae, Panopeidae and Portunidae larvae were concentrated in the mid and outer continental shelf since the freshwater plume “pushes” the larvae to locations away from the coast. The opposite was observed during the lowest outflow, these larvae concentrated near coastal areas (23 and 53 km from the coast) due to the lower freshwater outflow entering the ACS (Figs 6 and 7, S10 and S11 Tables).

Lines 331 – 334: I see support for Ocypodidae (Fig. 7A) dispersal from coast to offshore during period of greater plume discharge, but I do not follow the retraction comment. The abundance of this family just appears to be lower overall in October. Couldn’t this just be from seasonal changes in reproduction, rather than reduced plume flushing?

Answer: The Reviewer is correct, we agree. The abundance of this family is greater in the highest flow (May), when there is a reproduction peak. The inclusion of the paragraph: “Furthermore, few taxa were not affected by the Amazon plume. Leucosiidae larval density was higher on the mid continental shelf (158 km), while the density of Ocypodidae is also higher at 158 km in the highest flow (May), decreases in lowest flow period (October).”

Line 609: Again, “dispersion” or “dispersal”? Discharge more directly affects dispersal, but it is possible that the authors are commenting on distribution patterns (dispersion) of estuarine families.

Answer: Reviewer 2 is right. We are commenting on the distribution patterns (dispersion) of estuarine families. Thus, we kept “dispersion” on line 607, and “dispersal” elsewhere.

**Tables**

Table 1: Still includes “S” as a haul abbreviation for sub-superficial samples. This is confusing, as “S” is defined as salinity in the table’s caption.

Answer: The “SS” abbreviation was kept to identify the sub-surface samples, as it is actually not necessary to abbreviate the word salinity, as it causes confusion with the SS of the Surface Drag. We chose to keep salinity in full and abbreviate only the type of drag. S for salinity was kept only in table S12 as there is no mention of drag in this case.

Tables S10 and S11: Table S10 shows distance/location of larvae, while Table S11 shows seasonal changes. Are the larval counts (N and density) combined in these? In other words, are the seasonal densities in Table S11 total or mean values for all distances (Table S10)? This should be clearly defined in the table captions. Also, does “N” represent number of larvae collected? This should also be defined in the table captions.

Answer: S10 and S11 contain the same larval N, which corresponds to the total number of larvae collected by location (S10) and by month (S11). Thus, for the same group, for example Calappa sp. collected in the SS trawl throughout the study period, the total N is equal to 08 larvae, being (4+2+2) at 83km, 158km and 198km (S10) and (6+2) May and October (S11). In S10, the density corresponds to the sum of the density (and not the average because the data do not have a normal distribution, and the average is not a good estimator) at that location during the entire sampling period. In S11, on the other hand, it corresponds to the sum of all densities of each group in that particular month, regardless of location.

Table S12: I suggest adding the Table S12 caption to the supplemental document. Currently, it is only included in the manuscript text.

Answer: It was added.

We are immensely grateful for the careful revision by the reviewers, which has substantially improved our work.
